# Observation of the 1S–2P Lyman-α transition in antihydrogen

**DOI:** 10.1038/s41586-018-0435-1

**Published:** 2018-08-22

**Authors:** M. Ahmadi, B. X. R. Alves, C. J. Baker, W. Bertsche, A. Capra, C. Carruth, C. L. Cesar, M. Charlton, S. Cohen, R. Collister, S. Eriksson, A. Evans, N. Evetts, J. Fajans, T. Friesen, M. C. Fujiwara, D. R. Gill, J. S. Hangst, W. N. Hardy, M. E. Hayden, E. D. Hunter, C. A. Isaac, M. A. Johnson, J. M. Jones, S. A. Jones, S. Jonsell, A. Khramov, P. Knapp, L. Kurchaninov, N. Madsen, D. Maxwell, J. T. K. McKenna, S. Menary, J. M. Michan, T. Momose, J. J. Munich, K. Olchanski, A. Olin, P. Pusa, C. Ø. Rasmussen, F. Robicheaux, R. L. Sacramento, M. Sameed, E. Sarid, D. M. Silveira, D. M. Starko, G. Stutter, C. So, T. D. Tharp, R. I. Thompson, D. P. van der Werf, J. S. Wurtele

**Affiliations:** 1 Department of Physics, University of Liverpool, Liverpool, UK; 2 Department of Physics and Astronomy, Aarhus University, Aarhus, Denmark; 3 Department of Physics, College of Science, Swansea University, Swansea, UK; 4 School of Physics and Astronomy, University of Manchester, Manchester, UK; 5 Cockcroft Institute, Sci-Tech Daresbury, Warrington, UK; 6 TRIUMF, Vancouver, British Columbia Canada; 7 Department of Physics, University of California at Berkeley, Berkeley, CA USA; 8 Instituto de Fisica, Universidade Federal do Rio de Janeiro, Rio de Janeiro, Brazil; 9 Department of Physics, Ben-Gurion University of the Negev, Beer-Sheva, Israel; 10 Department of Physics and Astronomy, University of Calgary, Calgary, Alberta Canada; 11 Department of Physics and Astronomy, University of British Columbia, Vancouver, British Columbia Canada; 12 Department of Physics, Simon Fraser University, Burnaby, British Columbia Canada; 13 Department of Physics, Stockholm University, Stockholm, Sweden; 14 Department of Physics and Astronomy, York University, Toronto, Ontario Canada; 15 École Polytechnique Fédérale de Lausanne (EPFL), Swiss Plasma Center (SPC), Lausanne, Switzerland; 16 Department of Chemistry, University of British Columbia, Vancouver, British Columbia Canada; 17 Department of Physics and Astronomy, University of Victoria, Victoria, British Columbia Canada; 18 Department of Physics and Astronomy, Purdue University, West Lafayette, IN USA; 19 Soreq NRC, Yavne, Israel; 20 Physics Department, Marquette University, Milwaukee, WI USA; 21 IRFU, CEA/Saclay, Gif-sur-Yvette Cedex, France

**Keywords:** Exotic atoms and molecules, Experimental particle physics

## Abstract

In 1906, Theodore Lyman discovered his eponymous series of transitions in the extreme-ultraviolet region of the atomic hydrogen spectrum^1,2^. The patterns in the hydrogen spectrum helped to establish the emerging theory of quantum mechanics, which we now know governs the world at the atomic scale. Since then, studies involving the Lyman-α line—the 1S–2P transition at a wavelength of 121.6 nanometres—have played an important part in physics and astronomy, as one of the most fundamental atomic transitions in the Universe. For example, this transition has long been used by astronomers studying the intergalactic medium and testing cosmological models via the so-called ‘Lyman-α forest’^3^ of absorption lines at different redshifts. Here we report the observation of the Lyman-α transition in the antihydrogen atom, the antimatter counterpart of hydrogen. Using narrow-line-width, nanosecond-pulsed laser radiation, the 1S–2P transition was excited in magnetically trapped antihydrogen. The transition frequency at a field of 1.033 tesla was determined to be 2,466,051.7 ± 0.12 gigahertz (1*σ* uncertainty) and agrees with the prediction for hydrogen to a precision of 5 × 10^−8^. Comparisons of the properties of antihydrogen with those of its well-studied matter equivalent allow precision tests of fundamental symmetries between matter and antimatter. Alongside the ground-state hyperfine^4,5^ and 1S–2S transitions^6,7^ recently observed in antihydrogen, the Lyman-α transition will permit laser cooling of antihydrogen^8,9^, thus providing a cold and dense sample of anti-atoms for precision spectroscopy and gravity measurements^10^. In addition to the observation of this fundamental transition, this work represents both a decisive technological step towards laser cooling of antihydrogen, and the extension of antimatter spectroscopy to quantum states possessing orbital angular momentum.

## Abstract (web summary)

The observation of the 1S–2P Lyman-α transition in the antihydrogen atom, the antimatter counterpart of hydrogen, is reported.

## Main

Challenges to antimatter Lyman-α spectroscopy include the difficulty of fabricating optical components and continuous-wave^11^ or pulsed laser sources at these extremely short wavelengths, as well as the scarcity of anti-atoms. The current observation was made possible by a number of technical advances, including the development of a solid-state-based, pulsed Lyman-α source^12^, implementation of innovative plasma control techniques^13^, and the ALPHA Collaboration’s recent, marked improvement in antihydrogen trapping and accumulation or ‘stacking’ rate. Stacking provides a sample of several hundred anti-atoms^14^, accumulated over several hours. Taking advantage of a nanosecond-scale laser pulse and our low-background annihilation detection, we have also inferred information on the antihydrogen velocity distribution.

**Fig. 1:**
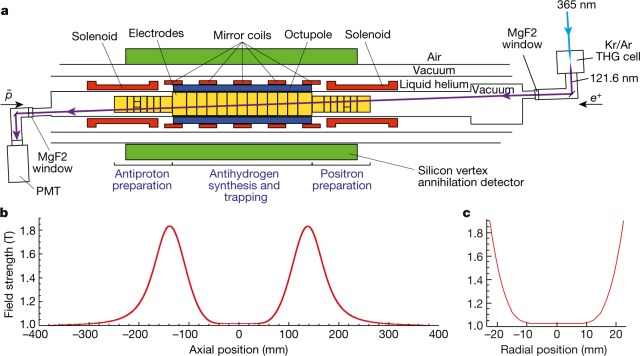
Experimental set-up. **a**, The three-layer silicon vertex annihilation detector is shown schematically in green; the external solenoid magnet for the Penning traps is not shown in this diagram. Laser light enters from the positron (*e*^+^) side (right) and is transmitted to the antiproton ($\bar{p}$) side (left) through vacuum-ultraviolet-grade MgF_2_ ultrahigh-vacuum windows. The laser beam crosses the trap axis at an angle of 2.3°. The transmitted 121.6-nm pulses are detected by a photomultiplier at the antiproton side. **b**, Axial magnetic well formed by the five mirror coils and responsible for the axial confinement of cold (less than 0.5 K) anti-atoms. **c**, Radial magnetic octupole field profile. PMT, photomultiplier tube; THG, third-harmonic generation.

Because matter and antimatter annihilate each other when they meet, antihydrogen must be created and then trapped in strong, inhomogeneous magnetic fields in an ultrahigh-vacuum chamber. The ALPHA-2 apparatus (Fig. 1a) is designed to combine antiprotons from CERN’s Antiproton Decelerator^15^ with positrons from a positron accumulator^16,17^ to produce and to trap atoms of antihydrogen. The techniques that we have developed to produce antihydrogen cold enough (below 0.54 K) to trap can be found elsewhere^13,14,18,19^. A typical trapping trial in ALPHA-2 involves mixing about 90,000 antiprotons and three million positrons to produce 50,000 antihydrogen atoms. Most of the antihydrogen atoms produced are either too energetic or in the wrong quantum state to be captured; typically 10–20 anti-atoms are trapped in one four-minute-long mixing cycle. We have employed the stacking technique referred to above to increase the total number of trapped antihydrogen atoms to several hundred for each measurement sequence.

The trapped anti-atoms are confined by the interaction of their magnetic moments with the inhomogeneous magnetic field, shown in Fig. 1b, c. The cylindrical trapping volume for antihydrogen has a diameter of 44.35 mm and an axial length of 280 mm. The magnetic field minimum of 1.033 T is near the centre of the trap, where the field is oriented axially. The field strength is flat to within one part in 10,000 in a 60 mm (axial) by 4.5 mm (radial) volume around the centre of the trap. The maximum field differential in the trap is 0.82 T along the axial direction and about 0.85 T along the radial direction.

**Fig. 2:**
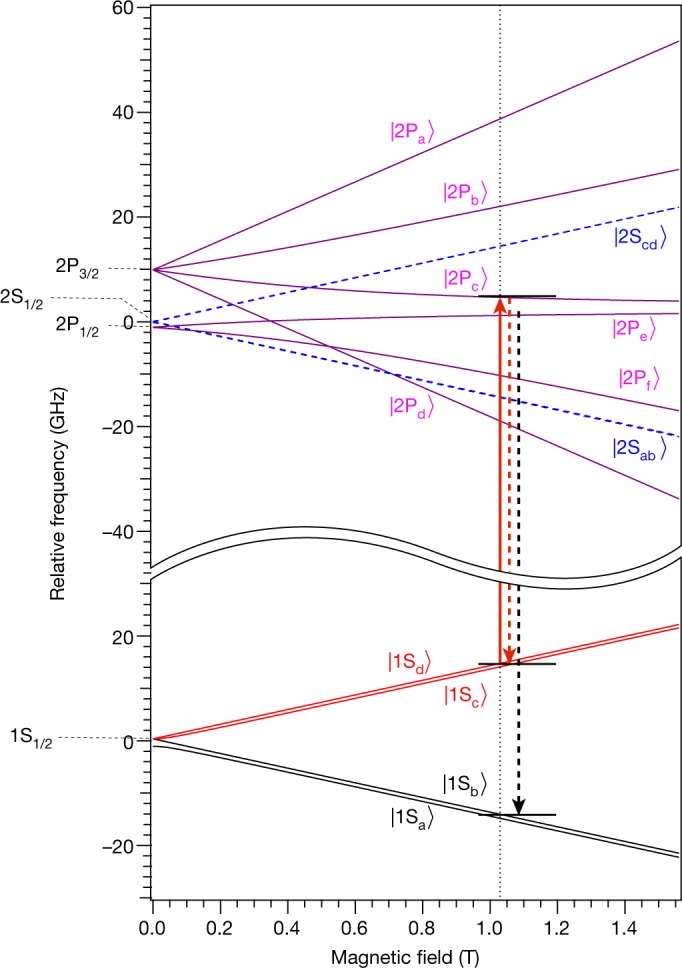
Trappable and untrappable energy levels in hydrogen. This plot reports the calculated energies for the sublevels of the 1S, 2S and 2P states for hydrogen as functions of the magnetic field strength. Note that the centroid energy difference *E*_1S–2S_ = 2.4661 × 10^15^ Hz is suppressed on the vertical axis. The vertical red arrow indicates the one-photon laser transition addressed here; the dashed red arrow illustrates the decay to the same trappable level, whereas the dashed black arrow corresponds to the decay to the untrappable level. Antihydrogen that decays to the untrapped 1S level escapes from the trap and can be detected by the annihilation detector.

Figure 2 shows the energy levels of hydrogen in the 1S and 2P states in a magnetic field. The 2S state is also depicted for reference. Antihydrogen atoms in the states labelled as 1S_c_ and 1S_d_ can be trapped. At zero magnetic field, the excited 2P state splits into two states (2P_3/2_ and 2P_1/2_) owing to the relativistic spin–orbit interaction. In non-zero magnetic fields, due to the Zeeman effect, the 2P_3/2_ state splits into four sublevels, while the 2P_1/2_ state splits into two. Each of these sublevels in turn splits into two states owing to the nuclear spin of the antiproton, but the splitting is much smaller (less than 0.1 GHz) than that in the ground (1S) state, and is therefore not visible on the scale of Fig. 2.

The 1S–2P_c_ transition studied here is a dipole-allowed transition. When antihydrogen is excited to one of the 2P sublevels, the excited state decays to the ground 1S state within a few nanoseconds by emitting a photon at 121.6 nm. Owing to the mixing of the positron spin states, there is a non-zero probability that the excited atom decays to either the 1S_a_ or 1S_b_ state instead of the original 1S_c_ or 1S_d_ state. Those that decay to the 1S_a_ or 1S_b_ state escape and annihilate at the trap walls and can be detected by the ALPHA-2 silicon vertex detector. The silicon vertex detector affords us single-atom detection capability, which is key to antimatter spectroscopy with few atoms^4,5^. The silicon vertex detector tracks the charged pions from the antiproton annihilation; the pion tracks are reconstructed to determine the location (vertex) of each annihilation. We employ machine-learning techniques, incorporating several parameters from the detector and the track reconstruction in a multi-variate analysis^20^ (MVA; see Methods), to distinguish antiproton annihilations from the cosmic ray background.

**Extended Data Fig. 1:**
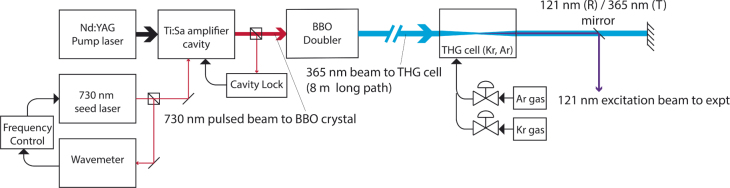
Laser system. The figure shows a schematic of the 121.6-nm laser system for driving the 1S–2P transition. See Methods for details.

In this experiment, the antihydrogen atoms were excited to the 2P_c_ state (Fig. 2) by light that was linearly polarized, the polarization vector being nearly perpendicular to the direction of the axial magnetic field. Narrow-line-width (about 65 MHz), pulsed (about 12-ns duration) laser radiation at 121.6 nm was used for the excitation. The system is described in detail in Methods, and the schematic diagram of the laser system is shown in Extended Data Fig. 1.

For each experimental sequence, about 500 antihydrogen atoms were accumulated in the trapping region by multiple stacking (typically 30 stacks) over an approximately two-hour period. Experience from past microwave manipulation experiments^5,6^ indicates that the 1S_c_ and 1S_d_ states are equally populated in the initially trapped sample. The trapped atoms were irradiated for about two hours by laser pulses using a 10-Hz repetition rate. During each two-hour sequence, the trapped atoms were exposed to radiation at twelve different frequencies around the calculated hydrogen resonance frequency of 2,466,051.625 GHz (using the average of the two 1S hyperfine levels). The magnetic field magnitude was determined by electron cyclotron resonance^21^. The frequency detunings ranged from −2.47 GHz to +0.85 GHz. The laser frequency was switched every 20 s in a pattern designed to minimize saturation and depletion effects (due to the finite population size) at each frequency. After each two-hour exposure, the remaining antihydrogen atoms were released and counted by ramping down the trap magnets in 15.6 s. We repeated the described sequence four times. The number of detected events is summarized in Table 1. With average laser pulse energies between 0.53 nJ and 0.65 nJ, about 60% of the trapped antihydrogen atoms were expelled and detected during the exposure time of two hours.

**Table 1:** Summary of experimental data

Sequence	Average pulse energy (pJ)	Number of pulses at each frequency	Total detected events during laser irradiation	Total detected events from the release of remaining atoms
1	620	6,000	230	170
2	620	6,000	254	164
3	650	6,000	261	179
4	530	6,000	221	174

The total number of antihydrogen atoms detected during the laser irradiation and during the release of remaining atoms is tabulated for each sequence. The MVA identifies annihilations with an efficiency of 0.807 for the laser irradiation and 0.851 for the release of the remaining atoms.

**Fig. 3:**
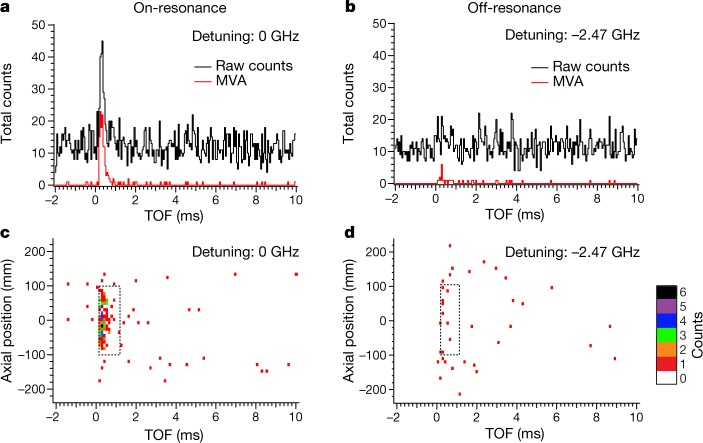
Temporal and spatial distribution of detected events. **a**, Temporal distribution of detected signals between −2 ms and 10 ms with respect to the laser pulse. The laser frequency was at the resonance of the 1S–2P transition (detuning 0 GHz). The black trace shows the raw detector triggers. The red trace shows events after background suppression due to an MVA-based machine-learning algorithm. The *y* axis is the total number of counts (bin size, 50 μs) for 24,000 pulses at the resonant frequency. **b**, Same as **a**, but the laser frequency was off-resonance; the detuning was −2.47 GHz. **c**, The axial position of the annihilation events is plotted versus the TOF. The detuning was 0 GHz. **d**, Same as **c**, but the detuning was −2.47 GHz. Events within the dashed box for each laser frequency were selected for the spectral shape determination (Fig. 4a).

Because the laser is pulsed, and the excitation light is present for about 12 ns for each pulse, the annihilation events due to the excitation are only expected to occur in the approximately 1-ms time window in which the untrapped atoms are forced to the trap wall. This greatly helps us to distinguish between signals due to laser-driven annihilations and those due to cosmic background. Figure 3 illustrates the power of the pulsed excitation method by comparing (1) on-resonance and (2) off-resonance measurements of the so-called time-of-flight (TOF) distribution. Even without the sophisticated machine-learning algorithm, raw detector triggers (Methods, Fig. 3a, black line) show a high signal level for the on-resonant case, in the time window between 0 ms and 1 ms after the laser pulse. The background is approximately constant. With the MVA (red line) the background almost disappears. In Fig. 3c, d we present two-dimensional plots for the axial position versus the TOF of the annihilation events. These plots also illustrate the selection criteria or ‘cuts’ (0 ms < *t* < 1 ms and −100 mm < *z* < 100 mm) used to accept events for the spectral shape determination described below. These cuts were predetermined on the basis of simulation information.

**Fig. 4:**
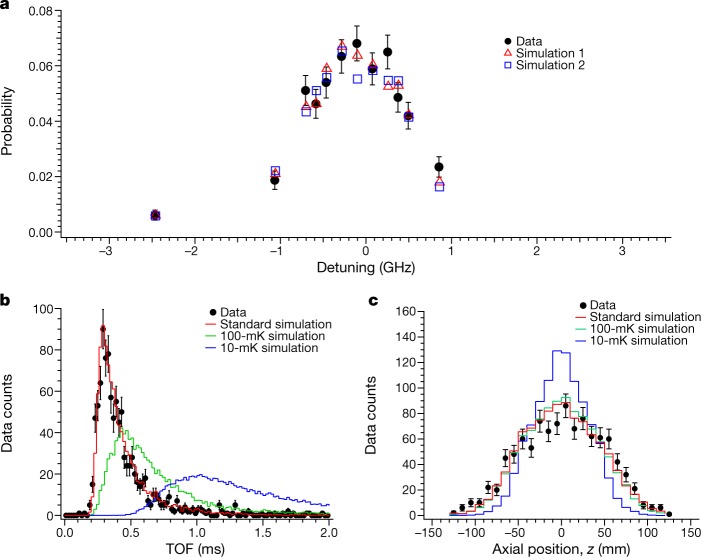
1S*–*2P line shape, time and axial distributions. **a**, The 1S–2P spectral line shape. Black, detected events, normalized to the total number of trapped antihydrogen atoms and scaled to the result obtained for a 600-s irradiation at 0.55 nJ. The error bars represent the statistical counting uncertainties. Red and blue, simulated line shapes for the initial conditions of *n* = 1 (simulation 1, red) and *n* = 30 (simulation 2, blue) (Methods). The statistical uncertainties of the simulation are within the size of each marker symbol. **b**, Black, the TOF distribution of all the detected annihilation events, measured relative to the laser pulse. Red, the simulated TOF distribution reflecting the experimental condition. Green and blue, simulated distributions for trapped antihydrogen atoms in thermal equilibrium at 100 mK and 10 mK, respectively. The simulated curves are normalized by area to the data (black). **c**, Comparison of the measured and simulated axial position distributions of the annihilations. The simulated distributions include the detector response function. Black, measured distribution. Red, simulated distribution. Green and blue, simulations with thermal distributions of 100 mK and 10 mK, respectively.

Figure 4a shows the spectral line shape of the transition, obtained using events with the above cuts applied. In this plot, the probability is normalized to the pulse energy; we have thus assumed that the excitation probability is linearly proportional to the laser pulse energy. The plotted data show that the transition from the 1S_c_ and 1S_d_ states to the 2P_c_ state has a line width of about 1.5 GHz (full-width at half-maximum, FWHM). If the hyperfine coupling constants of antihydrogen between the 1S and 2P states are the same as those for normal hydrogen, the spectrum in the magnetic field of 1.033 T would comprise two lines with a separation of 0.74 GHz. The two distinct lines are caused by the possible orientations of the antiproton nuclear spin in the 1S ground state. However, this hyperfine splitting is not resolved under the present experimental conditions owing to the Doppler broadening of the transitions caused by the motion of trapped antihydrogen atoms parallel with the laser beam. We note that the Doppler shift of the Lyman-α transition of hydrogen with a velocity of 75 m s^−1^ (characteristic for our magnetic trap depth) is 0.6 GHz. A fit to the measured data yields a resonant frequency of 2,466,051.7(0.12) GHz, in good agreement with the calculated hydrogen frequency quoted above. The contributions to the quoted uncertainty of 0.12 GHz are the wavemeter accuracy (0.06 GHz), the 730-nm laser (Methods) cavity lock stability (0.06 GHz), modelling uncertainties (0.07 GHz), and the statistical uncertainty in the curve fit (0.04 GHz). Many of these uncertainties may be reduced in the future, but the large natural line width of 2π × 99.6 MHz is an obstacle to using the Lyman-α transition for very high-precision measurements as tests of charge–parity–time-reversal (CPT) invariance.

The kinetic energy distribution of the trapped anti-atoms is also of interest. We expect, from previous measurements and simulations of the antihydrogen production and trapping processes^22^, that the trapped antihydrogen atoms are not in thermal equilibrium. Our standard model for antihydrogen formation is that the atoms are formed from antiprotons in equilibrium with the positron cloud, which has a temperature of about 20 K. The expected distribution of trapped atoms thus comprises the low-temperature tail of a Maxwellian distribution, truncated at the maximum trap depth of about 0.5 K. The measured line shape of the transition should thus be different from a Gaussian shape for a Doppler-broadened line. Therefore, we carried out detailed simulations (Methods), based on the known physics of hydrogen, to determine the expected line shape and intensity of the transition in our experimental environment. The simulated line shape is model dependent. Two examples of simulated results are shown in Fig. 4a in red and in blue. The calculated transition probability and line width match the data reasonably well over the region measured. The general agreement between the simulation and the observed spectral feature indicates that the observed line shape is indeed dominated by Doppler broadening, corresponding to an average energy of a few hundred millikelvin.

Figure 4b compares the time distribution of the detected signals following the laser pulse (black) to a simulated distribution (red) obtained using the standard formation model. The agreement between the simulation and the detected signal for these TOF distributions confirms that the radial velocity distribution of trapped antihydrogen is consistent with the model. For comparison, we have also simulated the TOF distribution of ejected atoms originating from hypothetical thermal distributions of 100 mK and 10 mK (Fig. 4b), which indicates that the detected time distribution should be sensitive to the radial temperature of trapped antihydrogen. In Fig. 4c we plot the measured axial distribution of laser-induced annihilation events, and we compare it to simulated results at various temperatures. These distributions also exhibit some dependence on antihydrogen temperature and may facilitate diagnosis of future laser cooling attempts.

In conclusion, we have reported here the observation of the 1S–2P, Lyman-α transition in antihydrogen, based on 966 detected events and an estimated background of 14 events. The frequency of this fundamental anti-atomic transition is determined to a precision of about 5 × 10^−8^, via narrow-line-width, nanosecond-pulsed, vacuum-ultraviolet laser spectroscopy. We also report a method of directly characterizing the kinetic energy of anti-atoms from their TOF to annihilation, following the laser-induced transition. These observations represent very important steps in the field of low-energy antimatter studies^23–27^. The techniques of optical manipulations and laser cooling, which have revolutionized the field of atomic physics over the past few decades, are about to be applied to anti-atoms. With its natural line width of about 100 MHz (comparable to our laser width), the Lyman-α transition can in principle be employed to cool antihydrogen to the 2.4-mK Doppler limit. Our simulations predict that cooling to about 20 mK is possible with the current ALPHA-2 set-up^9^. This, combined with other planned improvements, would reduce the 1S–2S transition line width by more than an order of magnitude and should eventually allow various other spectroscopic measurements with precisions approaching those achieved in hydrogen^28–30^. At such levels of precision, antihydrogen spectroscopy will have an impact on the determination of fundamental constants^31^, in addition to providing elegant tests of CPT symmetry. Furthermore, laser cooling will be crucial for a precision test of the weak equivalence principle via antihydrogen free fall^10^ or anti-atom interferometry^32^ at the 10^−2^ level and beyond. Access to the 2P state in antihydrogen greatly expands the future experimental horizon; for example, we will now be able to study fine-structure effects in an anti-atom.

## Methods

### Laser system for 121.6-nm light

The schematic diagram of the laser system is shown in Extended Data Fig. 1. The vacuum ultraviolet radiation at 121.6 nm is produced in two steps: frequency doubling of 730-nm pulses followed by third harmonic generation in a high-pressure gas cell^12^. The 730-nm pulses are produced by first seeding two titanium sapphire crystals with narrow line width (<100 kHz), continuous-wave radiation at 730 nm from a Toptica diode laser. The crystals are pumped by nanosecond pulses of the second harmonic of a Nd:YAG (neodymium-doped yttrium aluminium garnet) laser (Spectra Physics, pulse energy 300 mJ at 532 nm, with 10-Hz repetition rate). The generated 730-nm pulses have a pulse length and line width of 30 ns and 25 MHz, respectively. The 730-nm pulses are then converted to 365 nm by frequency doubling in a beta barium borate (BBO) crystal and then directed to the experimental zone. The third harmonic of 365 nm is generated in a high-pressure Kr/Ar gas cell (total pressure about 4 bar) after focusing the 365-nm pulses with an ultraviolet-grade lens (focal length 150 mm). The conversion efficiency from 365 nm to 121.6 nm is typically 10^−6^ to 10^−7^. The generated 121.6-nm pulses are collimated by a lens of 90-mm focal length and vacuum-ultraviolet-grade and then directed to the trap region by three vacuum-ultraviolet-grade mirrors placed in the vacuum system used to steer the light towards the antihydrogen trap.

Laser pulses enter the atom trap ultrahigh vacuum through an MgF_2_ window, which transmits about 65% of the incident light. The laser pulses cross the trap at an angle of about 2° to the direction of the trap axis, as shown in Fig. 1. Pulses exiting the trap are detected by a calibrated, solar-blind photomultiplier tube (Hamamatsu) to monitor the pulse energy and timing. The typical pulse width and line width at 121.6 nm are 12 ns and 65 MHz (FWHM, estimated). Each pulse has a pulse energy of 0.53–0.65 nJ in the trap region, and the repetition rate is 10 Hz, corresponding to an average power of 5–7 nW, and a peak power of 50–70 mW. The linear polarization is perpendicular to the direction of the magnetic field in order to drive the transition to the 2P_c_ state. The seed laser frequency is stabilized to a few megahertz by locking the laser to a wavemeter (HighFinesse). Before and after each experimental sequence, the frequency and line width of the 730-nm pulses were monitored by a custom-made, Fabry–Perot spectrometer having a free spectral range of 745 MHz, and a finesse of about 75.

### Suppression of cosmic ray background

To detect annihilation events in the two observation windows—(1) 15.6-s magnet ramp-down to release remaining anti-atoms and (2) 1-ms observation window after each laser pulse—we require different levels of background suppression. We optimized significance using an MVA-based, machine-learning algorithm to suppress unwanted background counts based on estimated populations of atoms in the apparatus per sequences.

The selection of variables has been described elsewhere^7^. The algorithm was trained and tested using a sample of 393,920 and 3,375,877 events for signal and background, respectively. The signal events were selected from periods with high rates of antihydrogen production in the apparatus, and thus contain less than 0.1% background. The background events were collected during periods when no antiprotons were present in the apparatus.

The trigger is configured to fire on the n-side of the ALPHA silicon hybrid^33^. The total configuration for the silicon vertex detector trigger requires more than two hits on the inner layer with one hit on the middle and outer layer.

#### The 15.6-s observation window

A classifier cut was chosen to optimize the significance for an expected 150 counts of signal and 320 counts of background (motivated to optimize significance of the signal when the laser is tuned to resonance in two sequences). The analysis gives a background rate of 0.217 ± 0.006 s^−1^ and an efficiency of 0.851 ± 0.002 (statistical error only, one standard deviation) annihilations per detector trigger.

#### The 1-ms (post-laser pulse) observation window

A classifier cut was chosen to optimize the significance for 31 expected counts of signal and 243 counts of background in the range ±10 cm along the beam axis of the trap centre. The analysis gives a background rate of 0.049 ± 0.003 s^−1^ and an efficiency of 0.807 ± 0.002 (statistical error only) annihilations per detector trigger. For the sample of 966 annihilation events reported here, the expected background is 14 events.

### Simulation of laser interaction with trapped antihydrogen atoms

The trapped antihydrogen motion is simulated as described elsewhere^34^ using the ***B***-field obtained from an interpolation of a Biot–Savart model of the ALPHA-2 mirror and octupole coils. The atoms are randomly distributed between the 1S_c_ and 1S_d_ states, which, for the purposes of the centre of mass motion, are treated as having the same magnetic moment, equal to that of an electron. To mimic the experiment, the simulated atoms are launched in the *n* = 30 state and allowed to radiatively cascade to the ground state, or are directly launched in the ground state to obtain two sets of initial conditions. The atoms are propagated for a few seconds before the laser is turned on, after which a laser pulse interacts with the atoms every 100 ms. At each laser pulse, the distance of the antihydrogen from the laser is computed and the laser intensity at that position is used to compute the excitation probability. The probability of the excited state decaying into the 1S_a_ or 1S_b_ states is also computed. A random number is compared to the product of these probabilities to determine whether the spin of the simulated atom flips for that laser pulse. If a flip occurs, the sign of the magnetic moment is reversed to give a high-field-seeking atom. The simulation is stopped when the position of the atom is outside the inner edge of the trap electrodes.

The statistics on properties like the Doppler width and the energy shift due to the Zeeman effect are accounted for by using the properties of the system at each laser pulse. The shift in frequency due to the Doppler effect is calculated from the velocity ***v*** at the time of the laser pulse:

$${\rm{\delta }}\omega =\frac{-\omega ({\boldsymbol{v}}\hat{k})}{c}$$

where $\hat{k}$ is the direction of propagation of the laser beam. The Zeeman shift for that laser shot is computed from the strength of the ***B***-field at the position of the antihydrogen. The 1S_c_ and 1S_d_ energies as a function of ***B*** are calculated as described in ref. ^34^, but the 2P energies are calculated using slightly more accurate equations which account for the difference between the positron magnetic moment and the Bohr magneton. The hyperfine splitting of the 2P states is at the level of a few tens of megahertz and, although included in the simulation, do not affect the results at the level presented here. The laser line width is included in the simulation by randomly shifting the laser frequency with a Gaussian distribution given by a FWHM of 2π × 65 MHz. These shifts are added to the detuning of the laser to obtain a total energy shift for that laser pulse of *ħ*δ*ω* relative to the line centre.

The probability that an excitation occurs during a laser pulse is proportional to the laser intensity at the position of the antihydrogen. The simulation assumes linearly polarized photons perpendicular to the direction of laser propagation, which is nearly parallel to ***B***. Thus, the polarization is nearly perpendicular to ***B***, and approximately half of the laser intensity can drive the transition. The transition dipole matrix element is equal to that of the *m* = 0 to *m* = 1 transition in hydrogen, multiplied by sin(*σ*) for the 2P_c_ level, where *σ* is a mixing angle; see equations (13) and (15) in ref. ^34^. The transition probability is multiplied by the Lorentzian factor from the natural line width of the 2P states:

$$\frac{{\left(\mathop{A}\limits^{\sim }/2\right)}^{2}}{{\left({\rm{\delta }}\omega \right)}^{2}+{(\mathop{A}\limits^{\sim }/2)}^{2}}$$

where $\mathop{A}\limits^{\sim }$ = 2π × 99.6 MHz is the natural line width of the 2P state, and δ*ω* is calculated as described in the previous paragraph. The probability of a spin flip in the decay is proportional to the probability in the 2P wavefunction with flipped spin: cos^2^(*σ*) for the 2P_c_ state.

### Data availability

The datasets generated and analysed during this study are available from J.S.H. on reasonable request.

## Online content

Any Methods, including any statements of data availability and Nature Research reporting summaries, along with any additional references and Source Data files, are available in the online version of the paper at 10.1038/s41586-018-0435-1.
